# SEM Evaluation of the Marginal Gap of Zirconia-Reinforced Lithium Silicate Full Crowns and the Effect of Post Crystallization: An In Vitro Study

**DOI:** 10.3390/dj12030061

**Published:** 2024-03-04

**Authors:** Asaf Shely, Diva Lugassy, Maxim Anufriev, Joseph Nissan, Olisya Rauchwerger, Gil Ben-Izhack

**Affiliations:** 1 Department of Oral Rehabilitation, The Maurice and Gabriela Goldschleger School of Dental Medicine, Sackler Faculty of Medicine, Tel Aviv University, Tel Aviv 6997801, Israel; asafshely@gmail.com (A.S.); nissandr@gmail.com (J.N.); dr.olisya@gmail.com (O.R.); 2 Department of Orthodontics, The Maurice and Gabriela Goldschleger School of Dental Medicine, Sackler Faculty of Medicine, Tel Aviv University, Tel Aviv 6997801, Israel; diva.lugassy@gmail.com

**Keywords:** CAD-CAM, CEREC (Chairside Economical Restoration of Esthetic Ceramic), Primescan, ZLS (zirconia-reinforced lithium silicate), marginal fit, marginal gap, marginal discrepancy, SEM

## Abstract

Background: This study compared the influence of crystallization on marginal gap adaptation by using computer-aided design and manufacturing (CAD-CAM) for producing monolithic zirconia-reinforced lithium silicate (ZLS) ceramic crowns. Methods: A total of 25 plastic teeth were scanned using a Primescan intra-oral scanner (IOS), and ZLS crowns were ground. For each unit (abutment and crown), the marginal gap was evaluated pre crystallization and post crystallization at four regions of interest through the use of a scanning electron microscope (SEM). To compare the marginal gap between the two groups, a Kolmogorov–Smirnov test performed on the study variables indicated a normal distribution (*p* > 0.05) followed by paired samples T-tests (α = 0.0005). Results: After crystallization, there were significantly higher circumferential marginal gaps (CMGs) for all four surfaces (distal (*p* = 0.0005), mesial (*p* = 0.0005), palatal (*p* = 0.0005), and buccal (*p* = 0.0005)). The total mean marginal gap (MMG) revealed a significantly higher result for the post-crystallization group (79.82 ± 7.86 μm) compared to the pre-crystallization group (24.25 ± 5.49 μm). Conclusions: The post-crystallization group showed a significantly higher marginal gap compared to the pre-crystallization group in all parameters, but both groups were in the clinically accepted threshold (<120 microns). In terms of the marginal gap, it is arguable whether to carry out post-crystallization for CELTRA^®^ DUO crowns and achieve better mechanical properties but significantly increase the marginal gap.

## 1. Introduction

In recent years, metal-free restorations have been accepted as a viable treatment option [1]. The need for a new restorative material with superior mechanical properties and esthetic performance has led to the introduction of CAD/CAM materials such as CELTRA^®^ DUO (Sirona Dentsply, Milford, DE, USA) [2]. CELTRA^®^ DUO (Denstsply Sirona, Charlotte, NC, USA), which is a type of zirconia-reinforced lithium silicate, also known as ZLS, is a relatively new ceramic material intended for CAD/CAM use. It consists of a dual microstructure: a homogeneous glassy matrix of lithium metasilicate (Li_2_SO_3_) and lithium orthophosphate (Li_3_PO_4_) with 10% zirconium dioxide (ZrO_2_) added to increase its mechanical characteristics (full material composition: 58% SiO_2_; 18.5% Li_2_O; 5% P_2_O_5_; 10.1% ZrO_2_; 1.9% Al_2_O_3_; 2% CeO_2_; 1% Tb_4_O_7_). Because of its high translucency and biaxial flexural strength values, it can be used for single partial and full restorations for anterior and posterior teeth along with occlusal veneers [1,2]. The flexural strength (three-point bending test), as reported in a recent study, of CELTRA^®^ DUO is 184.73 ± 13.63 (MPa) for LT ingots and 174.15 ± 21.76 (MPa) for HT ingots [3].

ZLS has two phases, the pre-crystallization phase and the post-crystallization phase, which are obtained with crystallization—a short heat treatment at 820 °C for 8 min [4]. In the pre-crystallized phase, lithium metasilicate and lithium orthophosphate was observed, and in the final crystallization phase, a new crystal phase of lithium disilicate was detected [2]. Computer-aided design (CAD) and computer-aided manufacturing (CAM) have become increasingly popular in dentistry over the past few decades. CAD/CAM can have many clinical applications such as inlays, onlays, crowns, and veneers [5]. Digital impressions including intraoral scanning can create a stereolithography (STL) file that can reduce time, cost, and space by eliminating the need for impression trays and materials [6].

One of the intraoral scanners (IOSS) that has been used in clinical practice since 2019 is Primescan (CEREC^®^ (Chairside Economical Restoration of Esthetic Ceramic) Primescan; Dentsply Sirona, Milford, DE, USA). Primescan uses confocal laser technology as opposed to triangulation scanning where light strips are projected on a 3D sample and recorded by a camera system. Confocal laser technology on the other hand uses a scanning technique where a microscope captures images by focusing on an optical light beam from a light source that passes through a specific lens making the mean light smaller and enhanced to scan more details. In addition, a change in the focus plane of the flat surfaces creates a 3D image that can minimize the distortions of the sample [7,8]. Diker et al. compared six different intraoral scanners and found that Primescan presented the highest accuracy among all scanners for a single crown preparation; Primescan had the best trueness (25 µm) and precision (10 µm) [9]. Dupagne et al. tested nine intraoral and extraoral scanners to evaluate their digitizing noise, dimensional trueness, and dimensional precision and showed that Primescan had the lowest digitizing noise (3.2 ± 0.6 µm) and the lowest precision error (7.6 ± 2.4 µm) [10].

Marginal gap accuracy in ceramic crowns is a major factor for a successful restoration; in the cases of marginal discrepancy, the restoration is prone to periodontal inflammation, cement breakdown, increased plaque retention, recurrent caries or pulp lesions, and bone resorption [11,12]. Today, although technology and materials have changed and there is more variation in the acceptable marginal gap [13,14,15], 120 µm, which was suggested by McLean and von Fraunhofer [16], is still considered the clinically acceptable limit of the marginal gap [17,18,19]. Many factors can affect the marginal gap, among them: the type of IOS, the milling or grinding system, the measurement method, the location and type of the finish line, the type of cement, the ceramic material type, and the preparation design [16,20,21]. Holmes et al., 1989, described the internal gap as a vertical line between the internal surface of the restoration to the axial wall of the preparation; the marginal gap is evaluated in the same way but is measured at the margins. Holmes also described absolute marginal discrepancy as a horizontal line from the margin of the restoration to the cavo-surface angle of the preparation [21]. Ng et al. found that when comparing the marginal fit of a single tooth restoration between digital (48 ± 25 µm) and conventional (74 ± 47 µm) methods, the digital method provided the better marginal fit [22].

Optical microscopes and a wide range of microscopy techniques have been used for dental practice and research for years, in particular scanning electron microscopy (SEM), with articles being published since 1962. SEM can be used in a variety of applications such as analyzing dental restorative materials, biomaterials, oral tissues, surface characteristics, the integrity of interfaces, and fracture analysis. In addition, SEM is based on non-invasive and non-destructive evaluation methods and shows high resolution and high magnification (×50 to ×5000) [23].

ZLS, as stated by manufacturers, can be used without the necessity of a crystallization firing process, which in turn reduces the working time [24]. Both D’Arcangelo et al. and Riquieri et al. determined that ZLS after the crystallization firing process demonstrated a higher flexural strength (from 170 MPa to 370 MPa). Riquieri et al. also found a decrease in size, the growth of lithium silicate grains, and higher hardness after crystallization [3,24,25].

We conducted a comprehensive review of the literature; we did not find any studies that compared the influence of post-crystallization on the marginal gap adaptation of zirconia-reinforced lithium disilicate (CELTRA^®^ DUO) single crowns. The null hypothesis was that no difference would be found between the pre-crystallization group and the post-crystallization group.

## 2. Materials and Methods

For this research, we used 25 maxillary right first molar plastic teeth (FLUX 8634; Columbia Dentoform, Lancaster, PA, USA); all teeth were prepared identically by the company (with a machine) as abutments for a single fixed partial denture with the following parameters: occlusal reduction 2.5 mm, 6 degrees convergence angle of the axial wall, and a finish line of 1.2 mm shoulder type. By using the IOS Primescan (CEREC^®^ Primescan; Dentsply Sirona, Milford, DE, USA), we scanned all 25 abutments, we received a virtual model (CEREC^®^ SW 5.2.4; Dentsply Sirona), and a single well-experienced user (G.B.I., 10 years of experience) marked the finish line for all 25 teeth. For the virtual design, the following parameters were set: radial spacer 90 µm, proximal contacts’ strength 25 µm, occlusal spacer 120 µm, dynamic contacts’ strength 25 µm, occlusal contacts’ strength 25 µm, radial minimal thickness 1000 µm, occlusal minimal thickness 1500 µm, margin ramp width 50 µm, margin thickness 50 µm, and margin ramp angle 60°.

**Figure 1:**
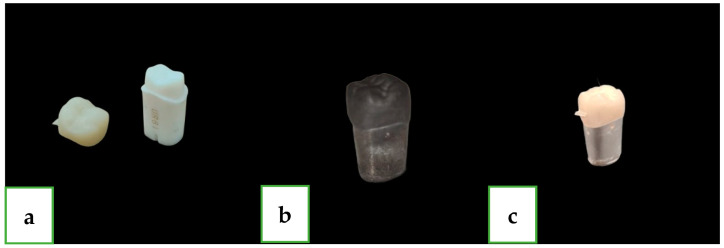
(**a**) First maxillary molar plastic tooth and zirconia-reinforced lithium silicate crown at the pre-crystallization phase: (**b**) unit (crown and abutment) at the pre-crystallization phase after coating and (**c**) unit at the post-crystallization phase before the secondary coating.

Twenty-five crowns made from zirconia-reinforced lithium silicate ceramic blocks (CELTRA^®^ DUO, Sirona Dentsply, Milford, DE, USA) were ground by using a 4-axes grinding machine (CEREC MC XL^®^; Dentsply Sirona) with two grits: Step Bur 12S and Cylinder Pointed Bur 12S (Dentsply Sirona, Charlotte, CN, USA) (Figure 1).

For creating a unit, which is defined as when the crown is cemented to the abutment, we used Temp-Bond (Temp-Bond™ NE™ Unidose; KaVo Kerr, Brea, CA, USA) for the cementation, and during the cementation process, we also used Lutron Fg-20kg Electronic Force Gauge (Lutron Electronic Enterprise Co., Ltd, Taipei City, Taiwan) which was set to 50 N.cm to apply a constant pressure until the cement reached the correct setting time, which is recommended by the manufacturer. 

**Figure 2:**
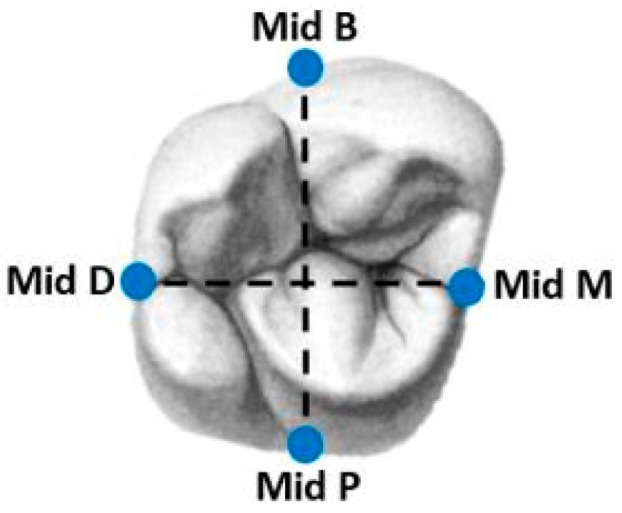
Four regions of interest for measurements at the middle of each side.

For the measurement process, we defined four sites in each unit (crown and abutment) at the following positions: mesial, buccal, distal, and palatal (Figure 2). For creating repeatable reference points, we used a new tungsten bur (FG330; Strauss&Co., Ra’anana Israel), and we drilled a hole in each of the four sides of the abutment (Figure 1). Before using the scanning electron microscope (SEM), we needed to complete another process, which was applying a gold coating (Figure 1). The process was carried out on each unit by using a mini sputter coater (SC7620; Quorum, East Sussex, UK) for 45 s.

**Figure 3:**
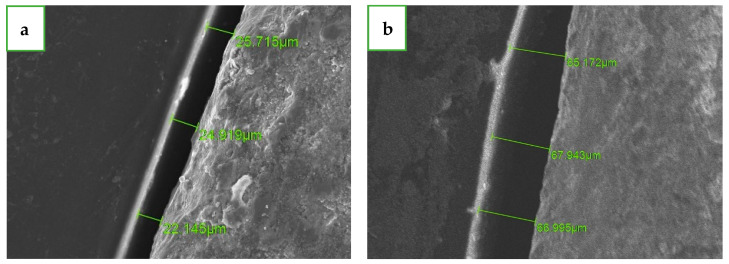
A side view measurement under the SEM. The average of the three green lines is the circumferential marginal gap (CMG). (**a**) Pre crystallization. (**b**) Post crystallization.

At the pre-crystallization phase, all 25 units were scanned using an SEM, we used a magnification of ×250 (JSM-IT100; JEOL, Akishima, Tokyo, Japan), and the same operator (M.A.) took the measurements by using operation software (InTouchScope™, https://www.jeol.co.jp/products/scientific/sem/JSM-IT500.html); three measurements were taken at the four reference points (regions of interest: B, M, P, and D), and the measurements were taken in the vertical dimension between the margins of the abutment and the crown, with a total of 12 measurements for each unit (Figure 3). The circumferential marginal gap (CMG) was defined and calculated as the average of the three measurements at each reference point. For each of the 25 units at the pre-crystallization phase, a total of 12 measurements were performed, and a total of 300 measurements were defined as a mean marginal gap (MMG).

After measuring all of the data for the pre-crystallization phase, we removed all of the crowns from the abutments, and the Temp-Bond was cleaned from the crowns and the abutments by using a steamer (Orix, Tel-Aviv, Israel). Then, the crowns were inserted into the furnace Programat^®^ CS2 (Ivoclar Vivadent Gmbh, Schaan, Liechtenstein) and went through the crystallization phase as recommended by the manufacturer (standby temperature: 500 Celsius; heating rate: 60 Celsius per minute; firing temperature: 820 Celsius; vacuum off).

The same rigorous measuring method was applied to all 25 units at the post-crystallization phase, as we described before.

We used the Statistical Package for Social Sciences for Windows Release 23.0 (SPSS Inc., Chicago, IL, USA) for statistical analysis of the results of this study.

To compare the marginal gap between the two groups, a Kolmogorov–Smirnov test performed on the study variables indicated a normal distribution (*p* > 0.05). A paired samples T-test (α = 0.0005) was used for the comparison of the MMG between the pre-crystallization group and the post-crystallization group for each surface (buccal, distal, mesial, and palatal) and for the total MMG. The statistical significance level for this work is *p* < 0.0005.

## 3. Results

The Kolmogorov–Smirnov test performed on the study variables indicated a normal distribution (*p* > 0.05).

**Figure 4:**
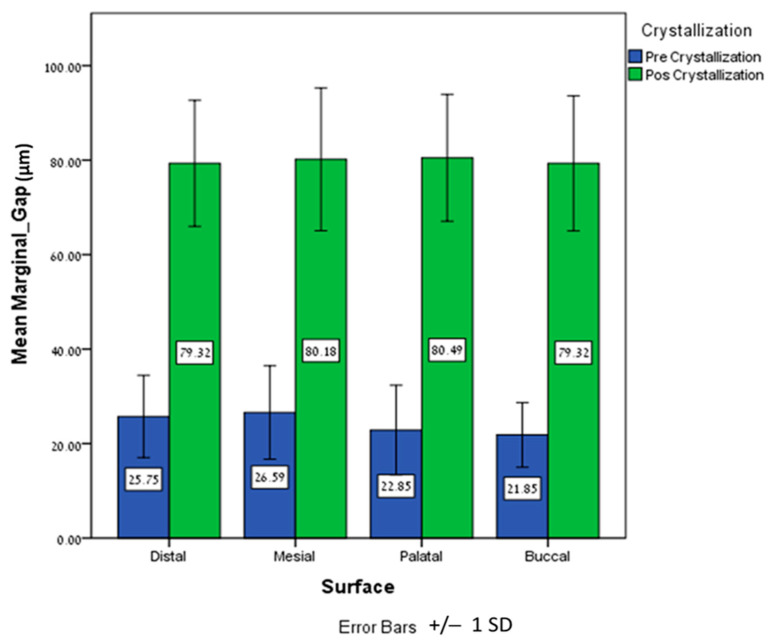
Mean and ±SD of the distal, mesial, palatal, and buccal CMGs (μm) pre crystallization and post crystallization.

Paired samples T-tests showed a significantly higher circumferential marginal gap (CMG) for the post-crystallization group on the distal (*p* = 0.0005), mesial (*p* = 0.0005), palatal (*p* = 0.0005), and buccal (*p* = 0.0005) surfaces compared to the pre-crystallization group (Table 1 and Figure 4).

**Figure 5:**
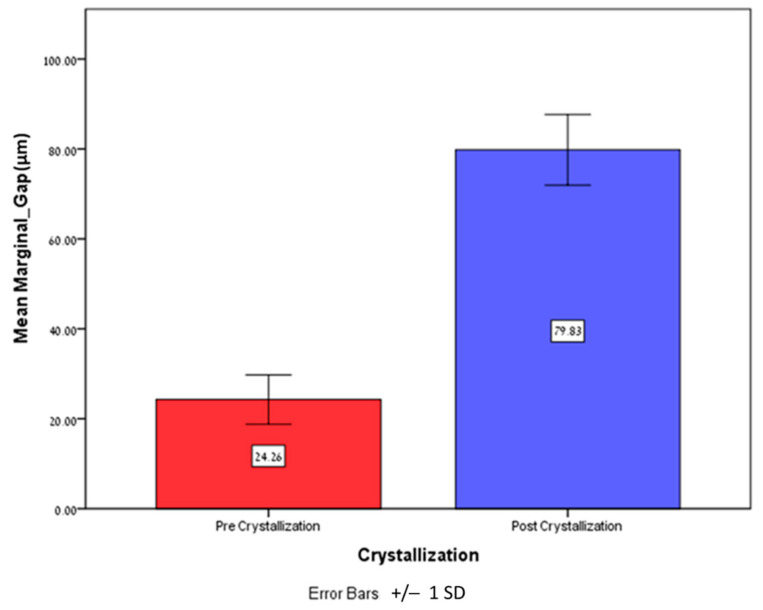
Mean and ±SD of the MMG (μm) pre crystallization and post crystallization.

The mean marginal gap (MMG) revealed a significantly higher marginal gap (*p* = 0.0005) for the post-crystallization group (79.82 ± 7.86μm) compared to the pre-crystallization group (24.25 ± 5.49μm) (Figure 5). The MMG of the post-crystallization group was more than three times higher compared to the pre-crystallization group. The lowest measurement was obtained in the pre-crystallization group (11.10 μm) and the highest measurement was obtained in the post-crystallization group (109.56 μm) (Table 2).

We rejected our null hypothesis as we found that there was a significant difference in the CMG and MMG between the two groups.

## 4. Discussion

In this in vitro research, our aim was to investigate how the crystallization process affects the marginal gap of ZLS CELTRA^®^ DUO (Denstsply Sirona, Charlotte, NC, USA) single crowns. We assumed that no significant difference would be visible. Our results indicate that our null hypothesis must be rejected as there were significant differences in the marginal gap between the two groups (pre crystallization and post crystallization) regarding all investigated surfaces (D, P, M, and B) and in total (MMG). As we have already mentioned, the marginal adaptation has a major influence on the success and survival of dental restorations; as the marginal gap increases cement dissolution may occur and it may lead to microleakage, increased plaque retention, and secondary caries, which eventually may cause pulp necrosis and bone resorption [11,12]. Our results demonstrate the significant impact of the post-crystallization process on the marginal gap of single zirconia-reinforced lithium silicate crowns: an increase in the marginal gap more than three times between the pre-crystallization and the post-crystallization process.

Zimmermann et al. compared the internal adaptation of CELTRA^®^ DUO (Denstsply Sirona, Charlotte, NC, USA) single crowns before and after crystallization. They used the PVS technique followed by a 3D technique with a subtractive method for calculating internal adaptation crowns (margin, axial wall, and occlusal surface). In their study, they stated that the post-milling (crystallization) process does not affect the internal adaptation of ZLS crowns (margin, axial wall, and occlusal surface). In our study, we received the opposite results as we showed a significant increase in the marginal gap after the post-crystallization process. We used a direct visualization technique (SEM) which may be more accurate than the 3D technique suggested by Zimmermann [26].

Many measurement methods are described in the current literature including the direct technique (an SEM or optical microscope) [27,28], the cross-sectioning technique [29], the impression replica technique [30], the radiographic technique [31], the micro-computerized technique [32], and quantitative evaluation [33]; each of the techniques has different advantages and disadvantages and may affect the results.

Schweitzer et al. examined the influence of different firing processes on the optical and mechanical properties of Celtra Duo and found that the firing process increases the flexural strength of the material but causes a decrease in volume. The decrease in the volume was referred to as the firing process [34]. In our study, the significant increase in the marginal gap could confirm that during the crystallization process, there is a three-dimensional change in the material, which affects the borders of the restoration where it meets the finish line of the preparation. We already know from previous studies that the crystallization process increases the marginal gap of glass–ceramic materials due to the densification shrinkage of the glass matrix [35,36]. Kim et al. found an increase of 12 μm between the pre- and post-crystallization groups when examining lithium disilicate crowns by using the silicone replica technique [35]. Gold et al. found an increase of 14 μm between the pre- and post-crystallization groups when examining lithium disilicate crowns by using the direct technique (optical microscope (500×)) [36]. In our study, we found an increase of 55 μm between the pre- and post-crystallization groups; this change could be due to the material as we used zirconia-reinforced lithium silicate, which has a different composition compared to lithium disilicate.

El-Ashkar et al. evaluated the marginal gaps of two different ZLS (VITA SUPRINITY and CELTRA^®^ DUO) materials by using three different techniques. For the CELTRA^®^ DUO group, they received a mean marginal gap of 86 μm when using the direct technique and 84 μm when using 3D superimposition analysis [37]. These results are similar to our results (80 μm) as, in both studies, we examined fully crystallized CELTRA^®^ DUO crowns. In our study, we compared the marginal gap of the same material pre crystallization and post crystallization, and in the study by El-Ashkar, they compared two different materials.

A recent study by Ferrini et al. evaluated the marginal gap of single crowns by using three different materials (lithium disilicate, zirconia, and a composite). Similar to our study, they used Primescan, measured each unit at twelve points, and evaluated the gap by using an SEM. Ferrini et al. used a metal abutment with a deep chamfer (2 mm) finish line. The zirconia exhibited the lowest marginal gap (21.45 ± 12.58 μm), followed by the composite (44.7 ± 24.96 μm) and finally lithium disilicate (62.28 ± 51.8 μm). The results of the zirconia are similar to the results of the pre-crystallization ZLS in our study (24.25 ± 5.49 μm), and the results of the lithium disilicate are similar to the results of post-crystallization ZLS in our study (79.82 ± 7.86 μm). As ZLS is a glass-based material with zirconia particles, it is very interesting that our results before and after crystallization are similar to zirconia and lithium disilicate, respectively [38].

Falahchai et al. examined four different preparations of natural teeth; in the second group, they made a 1 mm width rounded shoulder preparation, scanned it with the laboratory scanner 3Shape, fabricated ZLS (VITA Suprinity; VITA Zahnfabrik, BadSäckingen, Germany) crowns, and carried out a crystallization process. The crowns were cemented with Panavia (Panavia F2.0 white shade; Kuraray Noritake Denta, NY, USA), and measurements at a magnification of 202.9 were taken before and after cementation. Before cementation, they obtained 79.80 ± 9.97 μm, and after cementation, they obtained 115.80 ± 11.63 μm [39]. In our study, we obtained very similar results for the post-crystallization group: 79.82 ± 7.86 μm; although, we used another type of ZLS material, different finish line configurations, another type of scanner, and different measuring methods.

The marginal fit of restorations is affected by several factors, among them: the finish line configuration of the preparation; it is a highly investigated factor in the literature [40,41,42]. There are various studies regarding this issue, with some reporting that there is no significant difference between vertical and horizontal finish lines [27,43,44] and others reporting that a horizontal finish line (shoulder type) is more accurate than a vertical finish line regarding the marginal gap [45,46].

As we received a post-crystallization marginal gap of about 80 μm under ideal laboratory conditions, it is important to mention that we used Temp-Bond for cementation. In clinical practice where the conditions are less ideal, it is common to use adhesive resin cement, self-adhesive resin cement, or glass ionomer cement, which may increase the marginal gap by another 20–60 μm [39,47,48]; this may lead to a marginal gap of 120–140 μm, and such a marginal gap is beyond the acceptable clinical range (100–120 μm), which we know from the literature [16].

To our knowledge this is the first time that ZLS is being investigated before and after crystallization regarding the marginal gap; it is important to mention that more studies regarding this material need to be conducted with different methods, preparations, and cements regarding marginal and internal adaptation.

It is important to mention the limitations of this study. In the in vitro study, we used only one type of IOS, one type of material, one type of temporary cement (temp-bond)**,** and only one measuring technique (direct), and it was a static laboratory experiment.

Further studies are required to understand the influence of many other parameters on the marginal gap such as preparation parameters (finish line and convergence), digital settings (radial spacer and occlusal spacer), the wear of grits, and milling parameter variations.

## 5. Conclusions

With the limitations of this in vitro study, we suggest that:

1. The pre-crystallization phase (24.25 ± 5.49 μm) showed a significantly lower marginal gap compared to the post-crystallization phase (79.82 ± 7.86 μm) (*p* < 0.005).
2. Both the pre-crystallization phase and the post-crystallization phase were clinically acceptable (<120 microns).
3. Regarding the marginal gap, it is not recommended to crystallize CELTRA^®^ DUO.
4. It is arguable whether or not to perform post-crystallization for CELTRA^®^ DUO crowns and achieve better mechanical properties but increase the marginal gap significantly.
5. It is suggested to conduct future studies and use resin cement or glass ionomer cement to realize what is the true value of 80 microns in clinical conditions and whether it can cross 120 microns, which is the clinically accepted threshold.

## Figures and Tables

**Table 1:** Mean, ±SD, confidence interval (upper/lower limit), and minimum and maximum of the distal, mesial, palatal, and buccal CMG (μm) pre-crystallization and post-crystallization. (α = 0.0005).

	Distal Surface			Mesial Surface			Palatal Surface			Buccal Surface		
Marginal Gap (μm)	Mean ±SD	CI—Upper/ Lower limit	Min Max	Mean ±SD	CI—Upper/ Lower limit	Min Max	Mean ±SD	CI—Upper/ Lower limit	Min Max	Mean ±SD	CI—Upper/ Lower limit	Min Max
Pre crystallization	25.74 ±8.71	29.34 22.14	12.15 42.60	26.59 ±9.88	30.66 22.51	13.27 50.62	22.84 ±9.48	26.76 18.93	11.28 43.82	21.84 ±6.85	24.67 19.01	11.10 34.50
Post crystallization	79.31 ±13.35	84.83 73.80	53.40 101.07	80.18 ±15.10	86.41 73.94	55.39 106.70	80.49 ±13.44	86.03 74.94	59.29 109.56	79.32 ±14.29	85.22 73.41	51.76 106.52

**Table 2:** Mean, ±SD, confidence interval (upper/lower limit), and minimum and maximum of the MMG (μm) pre-crystallization and post-crystallization. (α = 0.0005).

Marginal Gap	Mean ±SD	CI—Upper/Lower Limit	Min Max
Pre Crystallization	24.25 ±5.49	26.52 21.99	16.01 38.54
Post Crystallization	79.82 ±7.86	83.07 76.58	65.02 103.03

## Data Availability

The data presented in this study are available on request from the corresponding author.
